# Medication Management Models for Polymedicated Home-Dwelling Older Adults With Multiple Chronic Conditions: Protocol of a Systematic Review

**DOI:** 10.2196/13582

**Published:** 2019-05-28

**Authors:** Filipa Pereira, Pauline Roux, Joëlle Rosselet Amoussou, Maria Manuela Martins, Armin von Gunten, Henk Verloo

**Affiliations:** 1 School of Health Sciences HES-SO Valais-Wallis Sion Switzerland; 2 Institute of Biomedical Sciences Abel Salazar University of Porto Porto Portugal; 3 Research Center for Psychology of Health, Aging and Sport Examination University of Lausanne Lausanne Switzerland; 4 Psychiatry Library Education and Research Department Hospital and University of Lausanne Lausanne Switzerland; 5 Higher School of Nursing of Porto Porto Portugal; 6 Service of Old Age Psychiatry Lausanne University Hospital Lausanne Switzerland

**Keywords:** medication-related problems, polymedication, older adults, frailty, home-dwelling, community-dwelling, models, clinical pathways, systematic review, protocol

## Abstract

**Background:**

Older adults with multiple chronic diseases commonly require complex medication regimes. When combined with frailty, cognitive impairment, and changing pharmacological prescriptions, older adults’ polymedication regimes increase the risk of medication-related problems (MRPs) and hospitalization. Effective, well-organized medication management could avoid MRPs and their clinical outcomes.

**Objective:**

Identify medication management models and analyze their impact on managing and preventing MRPs for polymedicated, home-dwelling older adults.

**Methods:**

We will conduct a systematic review of published articles in relevant professional scientific journals from inception until March 31, 2019, in the following electronic databases,: Embase; Medline OvidSP; PubMed (NOT Medline[sb]); Cumulative Index to Nursing and Allied Health Literature (CINAHL) EBSCO; PsycINFO OvidSP; Cochrane Library, Wiley; and Web of Science. We will also hand search the bibliographies of all the relevant articles found and search for unpublished studies. We will consider publications in English, French, German, Spanish, Italian, and Portuguese. Retrieved articles will be screened for eligibility. Statistical analyses will be conducted following the recommendations of the Cochrane Handbook for Systematic Reviews of Interventions, the Preferred Reporting Items for Systematic Reviews and Meta-Analyses (PRISMA), and Meta-analysis Of Observational Studies in Epidemiology (MOOSE) statements. Data will be analyzed using SPSS Statistics for Windows, version 25.0 (IBM Corp), and Review Manager, version 5.5 (The Nordic Cochrane Centre, The Cochrane Collaboration).

**Results:**

A preliminary search in Embase delivered 3272 references. This preliminary search allows us to complete our research strategy with equation development and to search the other databases. Relevant articles identified will allow for searching the reference lists for unpublished studies. The inclusion and exclusion criteria will be rigorously respected in the study selection. The entire study is expected to be completed by January 2020.

**Conclusions:**

This review will provide an exhaustive view of medication management models that could be effective for polymedicated, home-dwelling older adults and will allow us to analyze their impact on managing and preventing MRPs.

**Trial Registration:**

PROSPERO CRD42018117287; https://www.crd.york.ac.uk/prospero/display_record.php?RecordID=117287 (Archived by WebCite at http://www.webcitation.org/77fCfbCjT)

**International Registered Report Identifier (IRRID):**

DERR1-10.2196/13582

## Introduction

Health care systems across Europe are being challenged by an ageing population [1]. International statistics demonstrate that home-dwelling older adults consume huge amounts of prescribed and over-the-counter medication [2,3]. The risks of medication prescription and deprescription have been debated since the dissemination of systematic and umbrella reviews pointing out the inconsistency of some pharmacological treatments for older adults [4,5]. However, a substantial proportion of older adults have multiple chronic diseases requiring numerous treatment components and complex medication regimes [6]. Increasingly complex medication regimes, combined with frailty, reduced cognitive function, and changing pharmacokinetics and pharmacodynamics, increase the risk of adverse drug events and other medication-related problems (MRPs) in this population [7]. An MRP is “an event or circumstance involving medication therapy that actually or potentially interferes with desired health outcomes” [8]. MRPs include inappropriate prescribing (ie, wrong drug, dose, dosage frequency, or dosage form), drug interactions, adverse drug reactions, incorrect administration, the need for monitoring, and nonadherence to medication therapy [9]. MRPs occur frequently among polymedicated, home-dwelling older adults and are associated with increased risks of hospitalization, morbidity, and mortality [10-12]. Avoidable adverse drug events are the serious consequences of inappropriate drug prescribing [13]. For instance, adverse drug events alone contribute to 30%-40% of acute hospital admissions among older adults, although many are preventable [14]. The World Health Organization has estimated that 50% of patients suffering from chronic diseases either do not take their medication or fail to follow instructions for their medical prescription [15]. Medication management among polymedicated, home-dwelling older adults is a serious problem because of the increased burden of symptoms and disease, leading to the use of more medicines and a greater chance of suboptimal management. Estimates of medication nonadherence vary from 40% to 75% [16].

The misuse of categories of drugs such as sleeping pills, analgesics, tranquilizers, appetite suppressants, and stimulants is common [17]. Problems associated with low or zero therapeutic adherence are even more evident during the sensitive period following discharge home from hospital [18]. Suboptimal medication management may lead to a deterioration in the patient’s clinical condition, avoidable short-term hospitalizations or readmissions, physical and cognitive decline, exacerbated chronic medical conditions, and, consequently, increased health care use and costs [18-20]. The indirect impacts of the adverse effects of drug nonadherence, such as falls, dehydration, or delirium, may also lead to hospitalizations [21]. Older adults often undergo changes to dosage and prescribed medication during hospitalization [22] and during the first few months after hospital discharge due to comorbidities and the need for disease stabilization [19]. Such changes tend to decrease optimal medication management [23]. Older adults may also go back to taking medications that were discontinued during hospitalization, fail to begin a new treatment initiated during hospitalization, or take incorrect dosages [18]; they are particularly at risk of nonadherence in the first days or weeks after hospital discharge [18]. Home-dwelling older adults taking five or more drugs are considered to be more susceptible to the consequences of polypharmacy, such as adverse drug reactions, drug-drug interactions, nonadherence, or drug-food interactions. Monitoring, assessing, and reacting accordingly are requisite skills for optimal medication management in cases involving inappropriate polymedication or excessive polymedication [24]. Optimal medication management should thus be an integral part of older adults’ daily lives and is an essential condition for successfully maintaining them at home [25,26]. Moreover, taking into account the high prevalence of multiple chronic conditions in this population, optimal medication management often becomes a determinant of an older adult’s state of health and quality of life at home [27]. Medication management in polymedicated, home-dwelling older people has been described as the “single most important health care intervention in the industrialized world” [28]. This brings us to our search for the best practice models that optimize safety, the continuity of medication intake, and overall medication management among home-dwelling older adults [6]. With the overall aim of developing effective strategies to prevent MRPs and avoiding medication-related hospitalizations and rehospitalizations, many researchers and clinicians involved in primary care have tried to generate either general or specific structured, systematic, medication management models for polymedicated home-dwelling older adults.

One recent example of a structured action to prevent MRPs is the Medication Management Model. This is based on risk management and provides home-dwelling patients with medication review services comparable to those that benefit hospital and nursing home patients [29]. A second example concerns the Coordinated Medication Management Model, which involves home care nurses, nurses, physicians, and community pharmacists in medication processes for home-dwelling older adults, supported by home health care services [30]. Medication management models are interprofessional collaboration processes between patients, their informal caregivers, pharmacists, and nurses, based on evidence-based guidelines and bringing, if appropriate, identified problems to a physician’s attention [26]. To the best of our knowledge, there have been no systematic reviews summarizing the medication management models developed in community health care and their impact on MRPs among polymedicated, home-dwelling older adults.

## Methods

### Overview

This protocol has been registered in the International Prospective Register of Systematic Reviews (PROSPERO) (protocol number CRD42018117287). The systematic review will be conducted following the recommendations of the Preferred Reporting Items for Systematic Reviews and Meta-Analyses for Protocols (PRISMA-P) and its checklist for reporting harms [31,32], the reporting proposals of the Meta-analysis Of Observational Studies in Epidemiology (MOOSE) [33], and the methods outlined in the Cochrane Handbook for Systematic Reviews of Interventions [34].

### Purpose and Research Question

This systematic review’s purpose is to identify, examine, and summarize the models developed in community health care systems to optimize medication management for polymedicated, home-dwelling older adults. The following research question will guide this review: Which community health care models for optimizing medication management for polymedicated, home-dwelling older adults have been reported in interventional and observational studies?

### Inclusion and Exclusion Criteria

#### Types of Studies

This review will include randomized controlled trials, cluster randomized controlled trials, and nonrandomized studies. Nonrandomized studies will include quantitative studies examining the effects of medication management models that do not use randomization to allocate patients to comparison groups [34]. We will include retrospective and prospective epidemiological studies, cohort studies, case-control studies, controlled before-and-after studies, interrupted-time-series studies, and controlled trials with inappropriate randomization (ie, quasi-experimental studies) [35,36]. We will search for papers in French, German, English, Spanish, Italian, and Portuguese.

#### Types of Participants

This review will consider studies involving polymedicated, home-dwelling older adults with multiple chronic conditions and a minimum mean age of 65 years, as well as studies with participants aged 55 years or older. In order to properly include heterogeneity and complexity, we will consider multiple chronic conditions: the co-occurrence of at least two diseases in the same individual, cumulative indices considering both the number and severity of concurrent diseases, and the simultaneous presence of not only diseases but also symptoms and physical and cognitive dysfunctions.

#### Types of Models

We will examine all types of medication management models, including strategies, interventions, and clinical pathways aimed at optimizing the effects of medication management for polymedicated, home-dwelling older adults with multiple chronic conditions. Given their impact in reducing medication errors and enhancing interprofessional collaboration and patient safety, electronic medication management systems (EMMS) will be included in this study [37,38].

Where possible, these types of medication management models will be compared with usual care and will include strategies documented in models, interventions, and clinical pathways delivered by a primary health care provider alone or in collaboration with other allied health care professionals at home.

Based on the Effective Practice and Organization of Care (EPOC) taxonomy of health system interventions [39], we will consider medication management models, interventions, and clinical pathways targeting the health care professional level and the patient level, as discussed below, but we will exclude those targeting health care organizations:

1. Optimized medication management at the health care professional level:
  - Educational programs aimed at optimizing medication management.
  - Distribution of materials aimed at optimizing medication management.
  - Feedback to peers and other involved health care professionals on the effects and impact of medication management (ie, medication review from medical records).
  - Monitoring medication management models, including interventions and clinical pathways (ie, assessment, adjustment or change of medication, and medication deprescription).
  - Verbal recommendations to polymedicated, home-dwelling older adults by the health care providers involved to optimize medication management (eg, pharmacists and physicians).
  - The organized activities of teams for medication conciliation, prescription, and deprescription.
  - EMMS covering prescription, administration, pharmacy review, barcode medication administration, and anything that encompasses medication management processes for polymedicated, home-dwelling older adults with multiple chronic conditions [40].
  - Evaluations of the involvement of different health care professionals in the optimization of medication management.
2. Optimized medication management at the level of polymedicated, home-dwelling older adults:
  - Organized interventions aimed at optimizing medication management for polymedicated, home-dwelling older adults (ie, single- or multi-professional interactions conducted by nurses, pharmacists, or physicians, such as counselling on medication and medication compliance or patient education sessions).
  - Patient reminder systems aimed at optimizing medication management (ie, single- or multi-professional interventions conducted by nurses, pharmacists, or physicians, such as telephone contact and discharge planning; medication adherence aids, such as electronic monitors or pill dispensers; and meetings with the multi-professional health care team in the patient’s home).

#### Types of Outcome Measures

This review’s primary outcome measures will be:

1. The identification of models including interventions, clinical pathways, and EMMS aimed at optimizing medication management for polymedicated, home-dwelling older adults in primary health care.
2. The description of the components of the models, interventions, and clinical pathways and the identification of the stakeholders involved (ie, professional and nonprofessional caregivers).
3. The description of the impact of medication management models, interventions, and clinical pathways versus usual care on:
  - Rates of hospitalization for MRPs.
  - Rates of emergency department visits for MRPs.

Primary outcomes will be measured by different methods based on dichotomous (ie, yes or no), ordinal, or continuous rates and scores (eg, hospitalization or rehospitalization, frailty severity or progress, emergency department visits for MRP [10], and misuse of medication [41]).

This review’s secondary outcome measures will be descriptions of the associations between sociodemographic characteristics, health data, and MRPs (ie, nominal, ordinal, or interval level).

### Information Sources and Search Strategy

We will search the following databases, without restriction on the publication date: Embase (from 1947); Medline OvidSP (from 1946); a subset (sb) of PubMed (NOT Medline[sb]) (from 1996); Cumulative Index to Nursing and Allied Health Literature (CINAHL) EBSCO (from 1937); PsycINFO OvidSP (from 1887); Cochrane Library, Wiley (from 1992); and Web of Science (from 1900). Furthermore, we will search in the reference lists of relevant articles identified and for unpublished studies.

The search syntax will use Medical Subject Headings (MeSH) and text terms with Boolean operators. The syntax will consist of the search themes intersected by the Boolean terms “AND” and “OR.” MeSH terms and free keywords will include the following:

1. Terms for “Medication management,” “Drug therapy management,” “Therapeutic medication management,” and “Optimizing medication treatment.”
2. Terms for “Community-dwelling older adults,” “Home-dwelling older adults,” “Elderly,” “Aged,” “Home-care patients,” “Older adults,” and “Very old adults.”
3. Terms for “Home-dwelling,” “Living in place,” “Homebound,” “Primary care,” “Community health services,” “Community hospital,” “Ambulatory care,” “Outpatient clinics,” “Hospital,” “Ambulatory care facilities,” “Day care,” “Primary health care,” “Community health centers,” “Health services for the aged,” “Community,” “Domicile,” “Home or home-care or home-based,” “Outpatient,” “Day patient,” “Community care,” “Home-care services,” “General practice,” and “Urban population.”
4. Terms for “Multiple chronic conditions,” “Frailty,” “Vulnerability,” “Multimorbidity,” “Multimorbid,” “Muscle weakness,” “Fatigue,” “Slow motor performance,” “Low physical activity,” and “Unintentional weight loss.”
5. Terms related to “Drug-related problems,” “Medication-related problems,” “Misuse of medication,” “Medication abuse,” “Inappropriate polypharmacy,” “Polypharmacy,” “Excessive polypharmacy,” and “Polymedication.”
6. Terms for models, interventions, and clinical pathways: “Care model,” “Care map,” “Multidisciplinary care,” “Evidence-based care,” “Guideline,” “Patient care plans,” “Clinical paths,” “Clinical pathways,” “Critical paths,” and “Interventions.”
7. Terms related to “Medication management model,” “Medication optimization,” and “Reconciliation.”
8. Terms for “Computerized provider order entry,” “Electronic prescribing,” “Computer-assisted diagnosis,” “Computer-assisted therapy,” and “Medical device.”

### Study Selection

**Figure 1:**
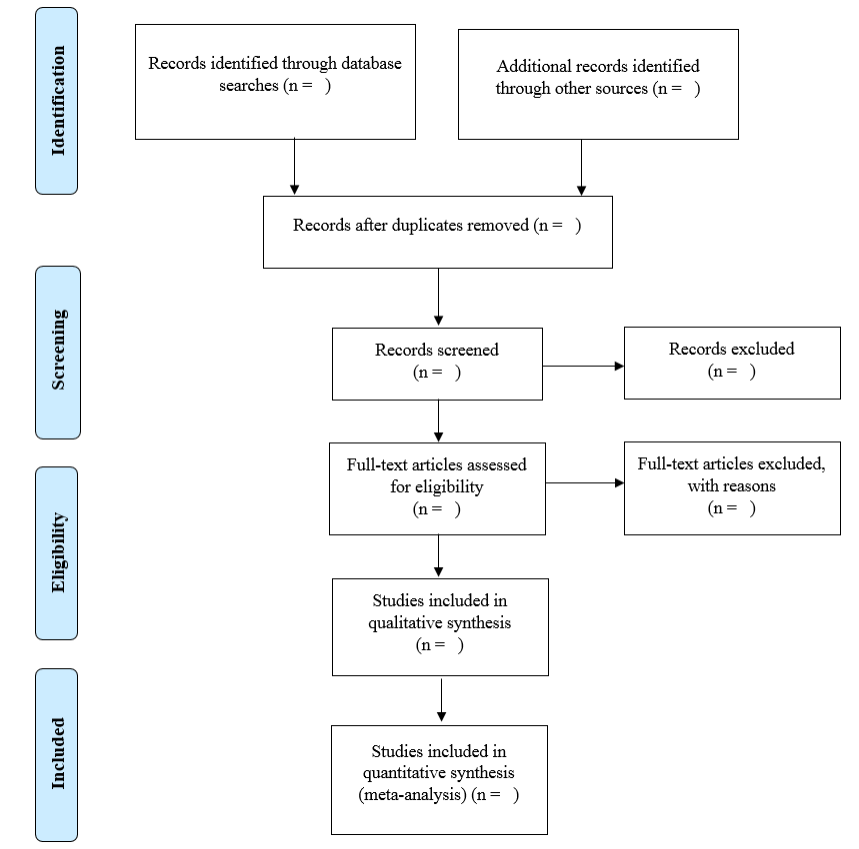
Blank flow diagram based on the Preferred Reporting Items for Systematic Reviews and Meta-Analyses (PRISMA) guidelines [42].

Two reviewers (FP and PR) will independently screen the titles and abstracts identified in the searches to assess which studies meet the inclusion criteria. Disagreements will be resolved through discussion or, if needed, a consensus will be reached after discussion with coauthors (MMM and HV). The reviewers will then independently assess the full-text articles to ensure that they meet the inclusion criteria. Disagreements will be discussed and resolved with coauthors (MMM and HV). A flowchart of the trial selection process has been drawn in accordance with the Preferred Reporting Items for Systematic Reviews and Meta-Analyses (PRISMA) statement [42] (see Figure 1).

### Data Extraction

Data will be extracted independently by two authors (FP and PR) using a specially designed, standardized data extraction form. Discrepancies will be resolved through discussion and consultation with coauthors (MMM and HV).

The following information will be extracted from each study included in the review: (1) study authors, year of publication, and country where the study was conducted; (2) study characteristics, including setting, design, duration of follow-up, and sample size; (3) participants’ characteristics, including age, sex, social status, marital status, educational status, level of autonomy, history of hospitalization and rehospitalization for MRPs, and emergency department visits for MRPs; medication management model; interventions; and clinical pathways; (4) multiple chronic conditions measured using indices (ie, the Charlson Comorbidity Index and the Cumulative Illness Rating Scale-Geriatric); and (5) types of outcome measures.

### Assessment of the Risks of Bias in Included Studies

Two reviewers (FP and PR) will independently assess the risks of bias in all the randomized and nonrandomized studies for interventions (NRSI) included, using the validated Cochrane Risk of Bias Tool, version 2.0 (The Cochrane Collaboration) [43]. This tool is based on five domains: (1) bias arising from the randomization process, (2) bias due to deviations from intended interventions, (3) bias due to missing outcome data, (4) bias in outcome measurement, and (5) bias in the selection of the results reported. Each of these five domains will be rated as (1) low risk of bias, (2) some concerns about bias, or (3) high risk of bias. Declaring that a study has a particular level of risk of bias in any individual domain will mean that the study as a whole has a risk of bias. Disagreements will be resolved through discussion and consultation with coauthors (MMM and HV).

We will also use the validated Risk of Bias In Nonrandomized Studies of Interventions (ROBINS-I) tool for assessing the risk of bias in NRSI [44]. This tool covers two dimensions and seven domains through which bias might be introduced into NRSI: (1) preintervention and during the intervention (ie, bias due to confounding, selection of study participants, or classification of the intervention) and (2) postintervention (ie, bias due to deviations from intended interventions, missing data, measurement of outcomes, and selection of the reported result) [44]. Any disagreements in quality assessments will be resolved through discussion.

### Statistical Analyses

Statistical analyses will be conducted by FP and PR following the recommendations of the Cochrane Handbook for Systematic Reviews of Interventions [34] and the PRISMA-P and MOOSE statements [31,33]. For dichotomous outcomes, average intervention effects (ie, pooled effect and meta-analysis) will be calculated as relative risks with 95% CIs using a random-effects model [45]. For continuous data, a random-effects model will be used to calculate weighted-mean differences with 95% CIs. If required, we will calculate standard deviations from the standard errors or 95% CIs presented in the articles. Heterogeneity will be quantified using the I^2^ and chi-square tests. Funnel plots will be drawn and Egger’s test will be computed to explore the possibility of publication bias [46].

Reasons for heterogeneity in effect estimates will be sought in meta-analyses [47,48]. To explore the possible determinants of heterogeneity, we will conduct subgroup analyses according to selected study characteristics (eg, participants’ ages; country where the study was conducted; types of professions; and types of models, interventions, and clinical pathways). Furthermore, sensitivity analyses will be conducted by (1) excluding relatively small studies (ie, fewer than 20 participants per randomization group) and (2) restricting analyses to good-quality studies. Data will be analyzed using SPSS Statistics for Windows, version 25.0 (IBM Corp), and Review Manager, version 5.5 (The Nordic Cochrane Centre, The Cochrane Collaboration).

## Results

To date, searches in Embase have been performed, delivering 3272 references. As of publication of this protocol, we are developing the search equations in the remaining databases to then initiate the process of study selection, rigorously applying the inclusion and exclusion criteria. The final results are expected in January 2020.

## Discussion

### Principal Considerations

To the best of our knowledge, this systematic review will be the first to synthesize evidence about medication management models, including their interventions, clinical pathways, and EMMS, as well as their impacts on MRPs among polymedicated, home-dwelling older adults.

Since MRPs are associated with an increased risk of hospital readmissions, morbidity, and mortality and are significant issues for the health care system, it is very important to develop intervention strategies to resolve or prevent them. The suboptimal medication management causing MRPs involving the hospitalization of polymedicated, home-dwelling older adults is underrecognized by community health care providers, especially by frontline community health care nurses. Therefore, an important task for community health care providers is to identify, resolve, and prevent the occurrence of MRPs in this rapidly growing population of polymedicated, home-dwelling older adults.

The results of this systematic review may guide future research in this avenue. The results may also contribute to the development of comprehensive and implementable recommendations for primary health care practitioners and policy makers concerning medication management among polymedicated, home-dwelling older adults.

### Strengths and Limitations

This systematic review protocol’s strengths are as follows: (1) clear definitions of the major concepts of medication management models, multiple chronic conditions, clinical pathways, and interventions; (2) the use of an appropriate search strategy designed in collaboration with a health librarian experienced in conducting such reviews; and (3) the inclusion criteria, which impose few restrictions on the study’s language, age, or geographic location. Nevertheless, there are several limitations that should be noted. The authors’ personal judgements may introduce bias into the assessment. Nonetheless, this risk will be reduced by using two reviewers to select and assess the eligibility of studies independently. Furthermore, it is possible that some eligible studies may not be covered by our research strategy. We will seek to minimize this limitation through the contribution of an experienced health librarian. Finally, the expected heterogeneity of studies about medication management models for polymedicated, home-dwelling older adults with multiple chronic conditions may influence our ability to state comprehensive and implementable recommendations from the literature.

### Conclusions

This systematic review will synthesize the available evidence about medication management models among polymedicated, home-dwelling older adults and their impact on MRPs. We expect our findings to provide meaningful evidence toward the optimization of health care models, programs, and services for polymedicated, home-dwelling older adults with multiple chronic conditions.

## Abbreviations

CINAHL: Cumulative Index to Nursing and Allied Health Literature
EMMS: electronic medication management systems
EPOC: Effective Practice and Organization of Care
MeSH: Medical Subject Heading
MOOSE: Meta-analysis Of Observational Studies in Epidemiology
MRP: medication-related problem
NRSI: nonrandomized studies for interventions
PRISMA: Preferred Reporting Items for Systematic Reviews and Meta-Analyses
PRISMA-P: Preferred Reporting Items for Systematic Reviews and Meta-Analyses for Protocols
PROSPERO: International Prospective Register of Systematic Reviews
ROBINS-I: Risk of Bias In Nonrandomized Studies of Interventions
sb: subset
